# Sublethal Effects of Four Insecticides with Different Modes of Action on Life History, Demography and Host Exploitation by the Egg Parasitoid, *Telenomus busseolae*

**DOI:** 10.3390/insects17050478

**Published:** 2026-05-07

**Authors:** Ahmet Bayram, Oğuzhan Doğanlar

**Affiliations:** 1 Department of Plant Protection, Agriculture Faculty, Dicle University, 21280 Diyarbakır, Türkiye; 2 Department of Medical Biology and Genetics, Medicine Faculty, University of Trakya, 22030 Edirne, Türkiye; doganlaro@yahoo.com.tr

**Keywords:** life table, population growth, Scelionidae, biological control, insecticide selectivity, *Sesamia nonagrioides*

## Abstract

Understanding how sublethal insecticide exposure affects natural enemies is crucial for sustainable pest management, yet such effects remain largely unexplored in some scelionid parasitoids, including *Telenomus busseolae* Gahan (Hymenoptera: Scelionidae). This study evaluated the compatibility of widely used insecticides with different modes of action—imidacloprid, indoxacarb, teflubenzuron, and chlorantraniliprole—with the biological control of the Mediterranean corn borer, *Sesamia nonagrioides* (Lefèbvre) (Lepidoptera: Noctuidae), by the egg parasitoid *T. busseolae*, a key natural enemy. Risk quotients (RQ), calculated as the ratio of field concentrations to LC_50_ (median lethal concentration) values, indicated low acute risk for all insecticides. Sublethal effects on adult parasitoids were assessed at LC_25_ concentrations using the age–stage, two-sex life table approach revealing significant impacts on longevity, fecundity, oviposition duration, and population growth parameters, including intrinsic rate of increase (*r*) and finite rate of increase (*λ*), with the most pronounced effects observed under imidacloprid exposure. Host exploitation remained mostly unaffected except under imidacloprid, which reduced net parasitism rate (*P*_0_), while the transformation rate (*Q*_p_ = *P*_0_/*R*_0_), representing the relationship between net parasitism (*P*_0_) and net reproductive rate (*R*_0_) at the population level, the stable parasitism rate (ψ), representing parasitism capacity under a stable age–stage structure, and the finite parasitism rate (ω), integrating parasitism capacity with population growth to describe overall parasitism potential, were unchanged. Population projections over 60 days (three generations) suggested the greatest decline under imidacloprid exposure. These findings demonstrate that even sublethal exposure can affect parasitoid fitness, host utilization, and population trajectories, emphasizing the importance of choosing insecticides that are selective for *T. busseolae* to maintain effective biological control.

## Simple Summary

Insecticides are widely used to control crop pests, but they can also affect beneficial insects that naturally help manage these pests. One such insect is the egg parasitoid *Telenomus busseolae*, which attacks the Mediterranean corn borer, a major pest of corn. Adult parasitoids exposed to the LC_25_ (lethal concentration killing 25% of the population) of four common insecticides—imidacloprid, indoxacarb, teflubenzuron, and chlorantraniliprole—exhibited sublethal effects, including reduced lifespan, fewer parasitized eggs, and impaired population growth, with imidacloprid causing the most severe effects. Their ability to exploit hosts was mostly unchanged, except under imidacloprid. These results show that exposure to concentrations that allow most insects to survive can subtly impair this parasitoid, reducing the long-term effectiveness in controlling an important pest of maize. Understanding these effects is important for developing farming practices that protect natural enemies, maintain pest control, and support more sustainable agriculture.

## 1. Introduction

Maize is one of the most economically important cereal crops worldwide, yet its production is severely threatened by insect pests. Among these, *Sesamia nonagrioides* (Lefèbvre) (Lepidoptera: Noctuidae) is a major multivoltine pest in the Mediterranean Basin and Africa, causing substantial damage through larval tunneling into stalks and cobs, with yield losses reported up to 100% under severe infestation [1,2,3,4]. Beyond yield loss, larval feeding reduces grain nutritional quality [3] and promotes mycotoxigenic fungi [5]. Because females lay eggs between leaf sheaths and stems—from which larvae quickly bore into stalks or cobs—insecticide effectiveness is greatly limited by their endophytic feeding habit [1,3], often necessitating multiple treatments throughout the growing season [3].

The egg parasitoid *Telenomus busseolae* Gahan (Hymenoptera: Scelionidae) is the most effective specialized natural enemy of *S. nonagrioides* [2,6,7], capable of achieving parasitism rates of 65–100% in fields with minimal pesticide use [6,7]. Its tolerance to cold storage for extended periods without significant fitness loss facilitates mass rearing and distribution, making it a practical candidate for augmentative biological control programs [8]. The integration of *T. busseolae* with chemical control therefore represents a promising IPM (integrated pest management) strategy [3,9]; however, the frequent application of broad-spectrum insecticides with different modes of action in maize agroecosystems raises concerns about their potential adverse effects on this key parasitoid [10,11].

Insecticides can have unintended lethal and sublethal effects on non-target parasitoids, impairing survival, reproduction, longevity, and host exploitation efficiency [12,13,14,15]. Although sublethal effects have been documented across several scelionid parasitoids, research on *T. busseolae* remains limited [10,11]. In *T. busseolae*, adult females exposed to LC_25_ concentrations (lethal concentration causing 25% mortality) of deltamethrin and cyfluthrin showed reduced parasitism efficiency and longevity [10], while preimaginal exposure significantly reduced the intrinsic rate of increase, total progeny, and net reproductive rate [11]. Similar sublethal impairments—including reduced emergence, skewed sex ratios, impaired host exploitation, and altered behavior—have been documented across *Trissolcus* and *Telenomus* species exposed to a range of insecticide classes [16,17,18,19]. Despite growing evidence, assessments have largely been confined to acute toxicity and single-generation parameters, leaving critical gaps in our understanding of population-level demographic responses for *T. busseolae* specifically [11].

Similarly, a large body of research has demonstrated that insecticides can adversely affect scelionid egg parasitoids, leading to reductions in survival, parasitism, emergence, longevity, and reproductive performance across multiple species, including *Trissolcus* and *Telenomus* spp. [20,21]. These effects have been reported across different insecticide classes such as organophosphates and pyrethroids [19,21], neonicotinoids and diamides [20,21,22,23], and botanical or bio-based compounds [21,24,25] and are often dose-dependent, with both lethal and sublethal impacts occurring even at sublethal exposure levels [19,24,25,26]. Collectively, these findings indicate that insecticide exposure can significantly disrupt key biological traits and reduce host exploitation efficiency in scelionid parasitoids.

The majority of previous studies on scelionid egg parasitoids have largely been confined to acute toxicity, toxicity classification, survival, and short-term biological parameters, leaving critical gaps in our understanding of population-level demographic responses. A holistic approach incorporating life-history traits, demography, and host exploitation efficiency—integrating both lethal and sublethal effects—is therefore critical for accurately predicting demographic consequences and developing effective and selective pest management strategies compatible with biological control agents. Life table parameters—including the intrinsic rate of increase (*r*), net reproductive rate (*R*_0_), mean generation time (*T*), finite rate of increase (*λ*), and population projection—provide comprehensive population-level assessments by simultaneously integrating age- and stage-specific survival and reproduction under sublethal exposure [14,27]. Complementing these demographic measures, host exploitation parameters such as the number of hosts attacked and the parasitism rate directly reflect the parasitoid’s biological efficiency in suppressing pest populations. Together, these metrics offer a holistic understanding of how sublethal insecticide exposure may influence the ecological performance, pest suppression capacity, and population-level demographic responses of parasitoids [27].

This study aims to evaluate the feasibility of combining four insecticides with distinct modes of action—chlorantraniliprole (diamide), imidacloprid (neonicotinoid), indoxacarb (oxadiazine), and teflubenzuron (benzoylurea)—with *T. busseolae* for suppression of *S. nonagrioides*. The insecticides were tested at LC_25_ concentrations, which fall below the 30% mortality threshold recommended for insecticide use, to maintain parasitoid efficacy IPM [12,20,21,22]. To address this, the sublethal effects of these insecticides on the life history of *T. busseolae* were examined using the age–stage, two-sex life table approach. Host exploitation (parasitism efficiency) and population projection trajectories were further assessed to reveal the parasitoid’s demographic responses and to determine the feasibility of integrating these insecticides with *T. busseolae* for effective pest suppression.

## 2. Materials and Methods

### 2.1. Telenomus busseolae Rearing

*Telenomus busseolae* was sourced from parasitized *S. nonagrioides* eggs collected in Diyarbakır, Türkiye, and reared in a controlled laboratory environment (26 ± 1 °C, 65 ± 5% RH, 16:8 L:D). The parasitoid culture was maintained on *S. nonagrioides* eggs under the same conditions. Both *S. nonagrioides* larvae and adults were continuously reared on specific diets developed by Giacometti [28], under the same environmental parameters (26 ± 1 °C, 65 ± 5% RH, 16:8 L:D). Oviposition was encouraged using cardboard cylinders wrapped with parafilm strips. Females deposited their eggs beneath the parafilm, which were then collected by unrolling the strips.

### 2.2. Insecticide Toxicity Bioassay and Lethal Concentration Determination

In this study, the four most commonly used insecticides in maize were examined. The active ingredients, trade names, concentrations, exposed time, and estimated lethal concentrations of the insecticides are presented in Table 1. The concentration–response mortality relationship of the insecticides on *T. busseolae* was established by exposing parasitoids to dry insecticide residue on filter paper (MN 640 m Macherey-Nagel, Duren, Germany, 110 mm diameter). The filter papers were dipped into five different concentrations per insecticide, plus a distilled water-only control, air-dried for 2 h under constant ventilation of a fume hood in laboratory conditions [10,11,12]. Concentration ranges were determined in preliminary bioassays to produce ≥ 80% mortality at the highest concentration (Table 2). To expose parasitoids (12–24 h old) to the insecticide-impregnated filter papers or control papers, cylindrical arenas made of transparent Plexiglas (40 mm internal diameter, 5 mm internal height) sandwiched between two glass plates (75 × 75 mm) were constructed and firmly held in place by clips. Three ventilation holes (3 mm diameter) were drilled through the wall of each arena to prevent a fumigation effect. One hole was used for the introduction of parasitoids and was later closed with a bamboo stopper. The other two holes were drilled on opposite sides of the lateral wall and covered with metallic mesh to prevent parasitoid escape. Clean air was allowed to pass through the two holes, using tubing connected to a pump. To ensure an accurate assessment of lethal concentrations, the wall and both bases of each arena were carefully covered with insecticide-treated or control filter paper. The wall was covered with a filter paper strip (5 × 126 mm) using double-sided adhesive tape, which was then drilled by a tiny pin to ensure ventilation through the lateral holes [10,12]. To prevent parasitoid mortality during bioassays, a drop of pure honey was applied to four different points of the side walls of the arenas.

Female parasitoids were mated for 12 h after emergence and then placed individually into glass vials (10 mm diameter, 35 mm high) with pure honey and kept for 12 h before the experiment. About 30–40 mated, fed female parasitoids (12–24 h old) were introduced carefully into each arena through the third hole, using a mouth aspirator, which was then closed with a bamboo stopper. Four replicates were carried out for each treatment, and the number of total parasitoids for each insecticide is presented in Table 2. The arenas were checked every hour and rotated to ensure parasitoid movement on the filter paper. After the exposure period, the arenas were gently opened, and all parasitoids (apparently dead or alive) were removed and singly transferred using a mouth aspirator to glass tubes (10 mm diameter, 35 mm high) containing the diet and kept under constant laboratory conditions. The number of dead and alive parasitoids was assessed 24 h after exposure to allow for the recovery of knocked-out individuals.

The lethal concentrations of pesticides were determined through probit analysis using SPSS (version 25). Estimated values were considered significantly different whether their 95% fiducial limits did not overlap [29].

To provide a comparative risk classification of the tested insecticides, risk quotients (RQ) were calculated for *T. busseolae* based on median lethal concentrations (LC_50_), which were determined via probit analysis, and field-recommended concentrations (FRC) of insecticides used for target pests (Table 1 and Table 2).

The risk quotient (RQ) was calculated as

RQ = FRC/LC_50_

LC_50_ values (mL/L) were converted to active ingredient equivalents (mg a.i./L) to ensure standardized comparison of toxicity (Table 2).

Risk categories were assigned according to Preetha et al. [30] as follows:

RQ < 50 = harmless; 50–2500 = slightly to moderately toxic; >2500 = toxic.

### 2.3. Life Table Experiments

*Telenomus busseolae* males and females (12–24 h old) treated with LC_25_ concentrations of each insecticide or with distilled water (control) were used to assess sublethal effects on life-history traits (Table 2). The exposure duration varied among insecticides due to differences in their modes of action. The neurotoxic insecticides imidacloprid and indoxacarb required 12 h under laboratory conditions to achieve ≥ 80% mortality. In contrast, the insect growth regulator teflubenzuron (a chitin biosynthesis inhibitor) and the ryanodine receptor activator chlorantraniliprole are slower-acting compounds; therefore, a longer exposure period of 24 h was used for these treatments. This approach ensured comparable biological effects across treatments despite differences in toxic dynamics. Following exposure to the LC_25_ concentration for the insecticide-specific durations determined from the lethal concentration bioassays (Table 2; 12 or 24 h depending on mode of action), one female and one male from the same treatment group were randomly paired and transferred to a glass tube containing a drop of pure honey. The pairs were maintained for 12 h to allow mating and feeding under controlled conditions (26 ± 1 °C, 65 ± 5% RH, and a 16:8 h light: dark photoperiod). Subsequently, 100 ± 5 fresh (<24 h old) host eggs (*S. nonagrioides*) affixed to cardboard strips were provided daily. Exposed eggs were incubated at 26 ± 1 °C, 65 ± 5% RH, and a 16:8 h L:D photoperiod until adult emergence. Emerging offspring were counted and sexed. Parasitized eggs that did not yield emerged parasitoids were dissected to determine developmental status and, where possible, the sex of non-emerged parasitoids.

Unemerged parasitoids were excluded from the age–stage, two-sex life table analysis, as they do not contribute to demographic parameters (e.g., fecundity, instrictic rate of increase, and sex ratio), but were included in the host exploitation (parasitism rate) analysis because parasitized hosts are removed from the host population regardless of parasitoid emergence.

The number of replicates in the life table experiments varied among treatments and is presented in Table 3. The initial cohort sizes were 40 mated pairs for the control and 25–30 mated pairs for the LC_25_ treatments, depending on the insecticide. Each replicate consisted of one male and one female from the same treatment, paired in a single glass tube. Replicates producing only male offspring (indicating unmated females) throughout their lifetime or involving loss of parasitoids during handling were excluded. After these exclusions, the final number of replicates for each treatment was 35 for the control, 28 for chlorantraniliprole, 26 for teflubenzuron, 25 for imidacloprid, and 24 for indoxacarb.

### 2.4. Data Analysis

#### 2.4.1. Life Table and Parasitism Rate Analysis

The raw data of *T. busseolae* individuals exposed to each insecticide LC_25_ concentration and for the control group, including data on longevity, survival rate (*l_x_*), offspring number per female (*R*_0_), and fecundity were analyzed according to the age–stage two sex-specific life tables theory [31,32,33]. The demographic traits and life-history fitness traits (*r*, *λ*, *R*_0_) were determined using the TWOSEX-MS Chart program [34,35]. Variances and standard errors were calculated using 100,000 bootstrap replicates [36], followed by a paired bootstrap test at a 5% significance level to compare all parameters between insecticide-treated and control cohorts [37].

Data on host egg parasitism (host exploitation) by *T. busseolae* females were analyzed following the method outlined by Chi and Yang [38] by using the CONSUME-MS Chart program [39]. For parasitism rates, the mean daily and total parasitism values for each females were calculated, along with the following parameters: the age-specific parasitism rate (*k_x_*), age-specific net parasitism rate (*q_x_*), and net parasitism rate (*P*_0_), which represents the mean number of parasitized eggs by an individual parasitoid over its lifespan; the transformation rate (*Q*_p_), which indicates the number of eggs required to produce a single offspring [38]; the stable consumption rate (ψ), which reflects the total parasitism capacity of a stable population normalized to a total size of one; and the finite consumption rate (ω), which integrates both the intrinsic rate of increase and parasitism rate [40].

#### 2.4.2. Population Projections

The population parasitism-rate projections were initiated with ten pairs of *T. busseolae*, all subjected to the same controlled experimental conditions for 60 days (equivalent to three generations of the parasitoid) to estimate the total population size. All projections were according to Chi [41] by using the TIMING MS Chart program [42]. All figures were generated using SigmaPlot version 12.5.

## 3. Results

### 3.1. Lethal Concentrations

Probit analysis indicated that the LC_25_ values for *Telenomus busseolae* were 0.114, 0.421, 1.868, and 0.398 mL/L for imidacloprid, indoxacarb, teflubenzuron, and chlorantraniliprole, respectively (Table 2). Imidacloprid and indoxacarb caused 80–100% mortality after 12 h of exposure, whereas teflubenzuron and chlorantraniliprole required 24 h at the highest tested concentrations to reach similar mortality levels. The concentration–response models showed good fit, as indicated by non-significant chi-square goodness-of-fit tests (*p* > 0.05; Table 2).

All LC_25_ values were below the respective field-recommended concentrations (Table 1 and Table 2); 0.114 and 0.421 mL/L compared with 1.75 and 1.5 mL/L for imidacloprid and indoxacarb, and 1.868 and 0.398 mL/L compared with 2 and 0.75 mL/L for teflubenzuron and chlorantraniliprole, respectively.

The calculated RQ values for all four insecticides were <50, indicating that field-recommended concentrations for lepidopteran pests, specifically *S. nonagrioides*, are considered harmless to *T. busseolae*; however, LC_25_-based bioassays revealed significant sublethal effects on the parasitoid’s life table parameters, including reductions in longevity, fecundity, and intrinsic rate of increase.

### 3.2. Effects of Insecticides on Life-History Traits

Exposure to LC_25_ concentrations of insecticides significantly influenced the life-history traits of *T. busseolae* (Table 3). Total fecundity was highest in parasitoids exposed to chlorantraniliprole (118.3 eggs), which did not differ significantly from the control (123.1 eggs). In contrast, parasitoids exposed to teflubenzuron (97.5 eggs) and indoxacarb (89.9 eggs) exhibited reduced fecundity, while those exposed to imidacloprid produced the fewest offspring (72.9 eggs).

Female offspring production decreased significantly across all insecticide treatments compared with the control (69.8 eggs), with imidacloprid causing the greatest reduction (49.9 eggs). Male offspring production was highest following chlorantraniliprole exposure (65.1 eggs), exceeding the control (53.2 eggs), and lowest in parasitoids exposed to imidacloprid (23.0 eggs), indoxacarb (31.1 eggs), and teflubenzuron (40.8 eggs) (Table 3).

Net reproductive rate was calculated separately for female (*R*_0_), male (*R*_0_) offspring, and total offspring (*R*_0_). The net female reproductive rate (*R*_0_) of parasitoids remained statistically comparable among all treatments ranging from 24.9 to 34.9 female offspring per individual. However, the net male reproductive rate was significantly reduced in parasitoids exposed to LC_25_ of indoxacarb (15.5) and imidacloprid (11.5), compared to the control (26.6). Total net reproductive rate (*R*_0_) was highest in the control (61.5) and chlorantraniliprole (59.2), and lowest following imidacloprid exposure (36.4) (Table 3). Relative to the control, total *R*_0_ was reduced under all insecticide treatments, with reductions of 3.9% (chlorantraniliprole), 20.8% (teflubenzuron), 27.0% (indoxacarb), and 40.8% (imidacloprid). The intrinsic rate of increase (*r*) and finite rate of increase (*λ*) were also significantly reduced only in parasitoids exposed to LC_25_ of imidacloprid (*r* = 0.2032; *λ* = 1.2253) compared to the control (*r* = 0.2281; *λ* = 1.2557) (Table 3).

Oviposition duration varied significantly among the treatments. Parasitoids exposed to chlorantraniliprole maintained longest oviposition days (9.2 days), comparable to the control (8.6 days). In contrast, teflubenzuron (6.6 days), indoxacarb (5.7 days), and imidacloprid (3.7 days) progressively reduced oviposition duration (Table 3).

Adult longevity was strongly affected by insecticide exposure. Female longevity was significantly extended by chlorantraniliprole (20.7 days) and teflubenzuron (20.3 days) compared to the control (15.8 days); however, it was markedly reduced by indoxacarb (9.5 days) and imidacloprid (8.8 days). Male longevity followed a similar pattern, with values of 17.4 days (teflubenzuron), 16.0 days (control), 13.9 days (chlorantraniliprole), 9.5 days (indoxacarb), and 8.7 days (imidacloprid). Sex-specific differences in *T. busseolae* longevity were significant only in parasitoids exposed to chlorantraniliprole only, where females outlived males (Table 3).

On the other hand, the emergence rate of eggs parasitized by *T. busseolae* exposed to LC_25_ concentrations of the four insecticides was not significantly affected by parental exposure (F = 1.641, df = 4, 133; *p* = 0.168), indicating that sublethal insecticide treatment did not influence offspring viability. In contrast, the sex ratio (% female) of the offspring generation varied significantly among treatments, with a male-biased sex ratio observed in parasitoids exposed to chlorantraniliprole (F = 11.39, df = 4, 133; *p* < 0.001). Indoxacarb and imidacloprid treatments produced the highest female-biased progeny, whereas Teflubenzuron and the control exhibited intermediate proportions of females (Table 3).

**Figure 1:**
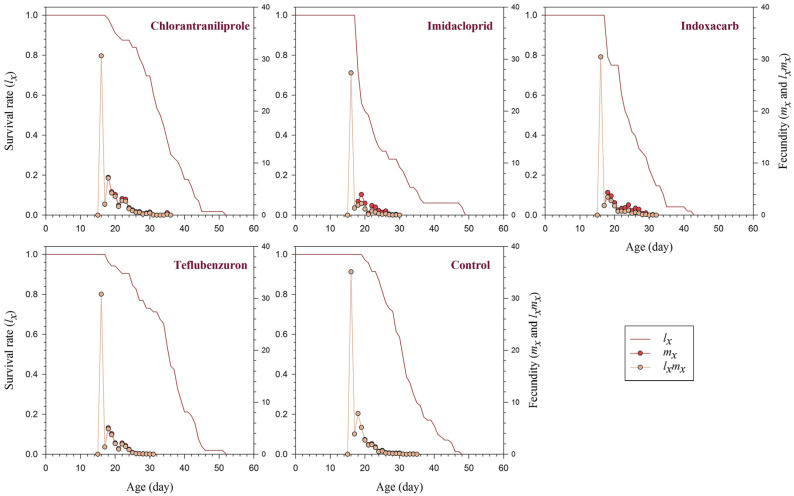
Age-specific survival rate (*l_x_*), age-specific fecundity (*m_x_*), and age-specific net maternity (*l_x_m_x_*) of *Telenomus busseolae* adults exposed to LC_25_ concentration of four different insecticides and a water control.

The survival and fecundity data of *T. busseolae* exposed to LC_25_ concentrations of the insecticides revealed significant differences in their life-history traits. The first 16 days are preimaginal, occurring within the host eggs. For chlorantraniliprole-treated parasitoids, survival rates (*lx*) began to decline shortly after the third day (day 18) (0.98). In the imidacloprid treatment, survival decreased sharply on day 18 (0.72), followed by indoxacarb (0.79) and teflubenzuron (0.96). In contrast, in the control treatment, the first adult mortality was recorded on day 20, with a survival rate of 0.97. The fecundity (*mx*; female offspring per female) of *T. busseolae* was highest on the first day of parasitoid (day 16) in all treatments. The mean fecundity values on the first day for chlorantraniliprole, imidacloprid, indoxacarb, teflubenzuron, and the control were 30.6, 27.3, 30.4, 30.7, and 35.0, respectively (Figure 1).

**Figure 2:**
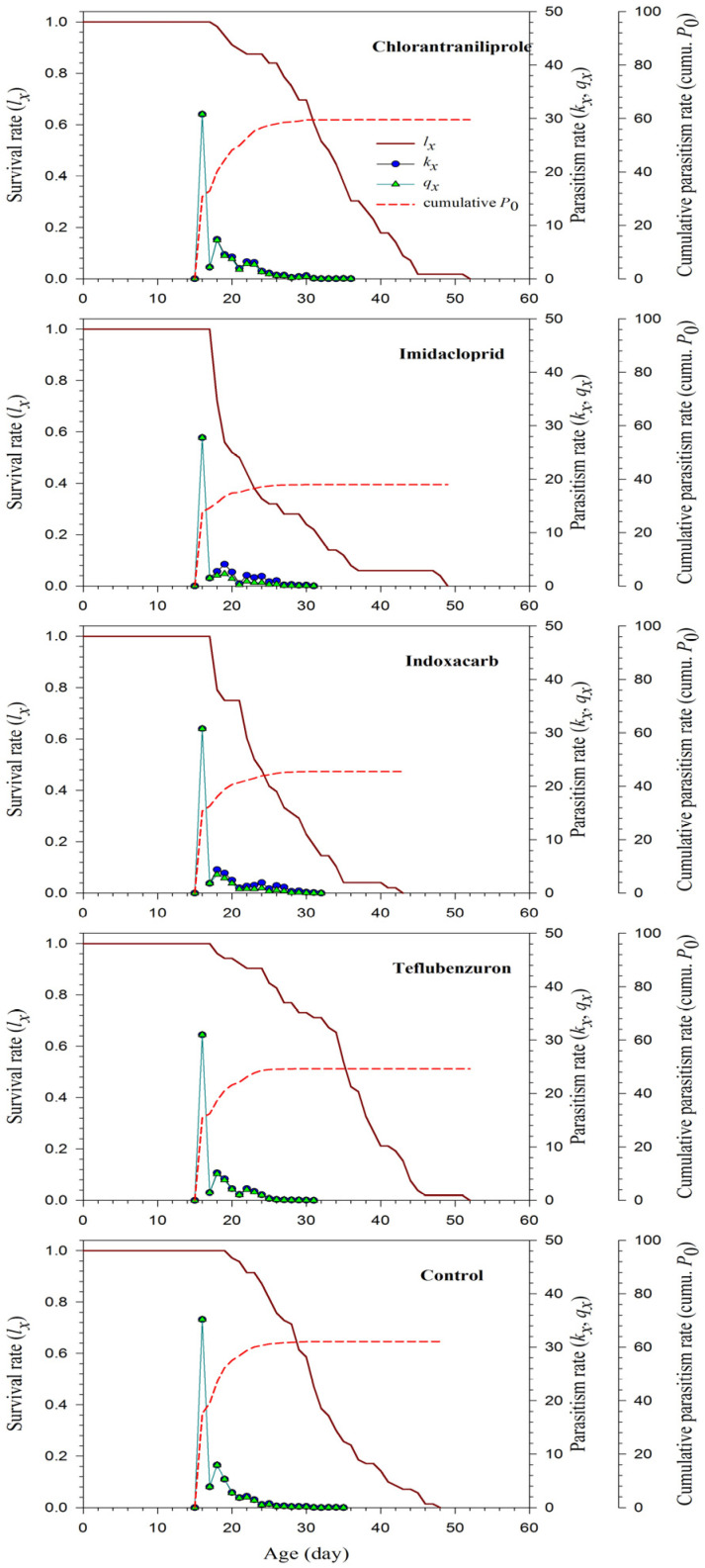
Age-specific survival rate (*l_x_*), age-specific parasitism (host exploitation) rate (*kx*) and age-specific net parasitism rate (*q_x_*), and net parasitism rate (cumulative *P*_0_) of *Telenomus busseolae* adults exposed to LC_25_ concentration of four different insecticides and a water control.

In line with these findings, analysis of age-specific parasitism (*kx*), age-specific net parasitism rate (*qx*), and net parasitism rate (*P*_0_) in relation to survival rates showed that peak parasitism (*kx* and *qx*) occurred on the first day of adult emergence of *T. busseolae* in all treatments. Following this initial peak, both *kx* and *qx* decreased progressively with advancing age, with minimal parasitism observed several days before the death of the last adult *T. busseolae* (days 52, 5, 43, 5, and 48 for chlorantraniliprole, imidacloprid, indoxacarb, teflubenzuron, and the control, respectively). Insecticide-treated and untreated *T. busseolae* females reached their peak cumulative net parasitism rates (cumulative *P*_0_) on days 36, 30, 31, 32, and 35, with values of 59.6, 37.9, 45.5, 49.2, and 62.1, respectively. No further parasitism was detected in any treatment after these time points (Figure 2). Cumulative net parasitism (cumulative *P*_0_) was highest in the control treatment and the lowest in the imidacloprid and indoxacarb treatments (Figure 2).

**Figure A1:**
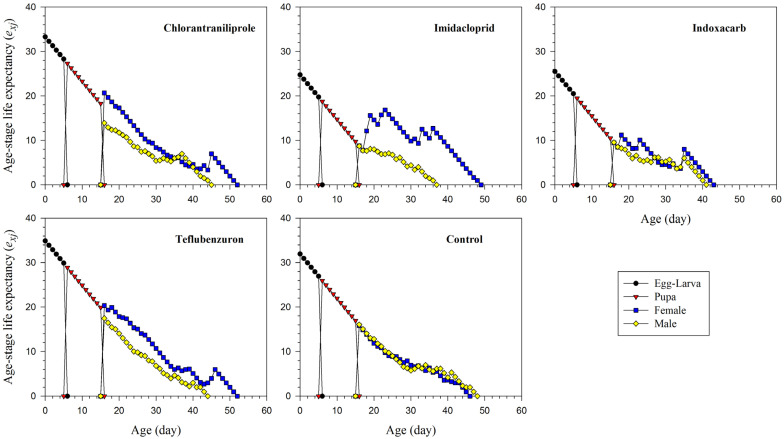
Age–stage life expectancy (*E_xj_*) of *Telenomus busseolae* adults exposed to LC_25_ concentration of four different insecticides and a water control.

**Figure A2:**
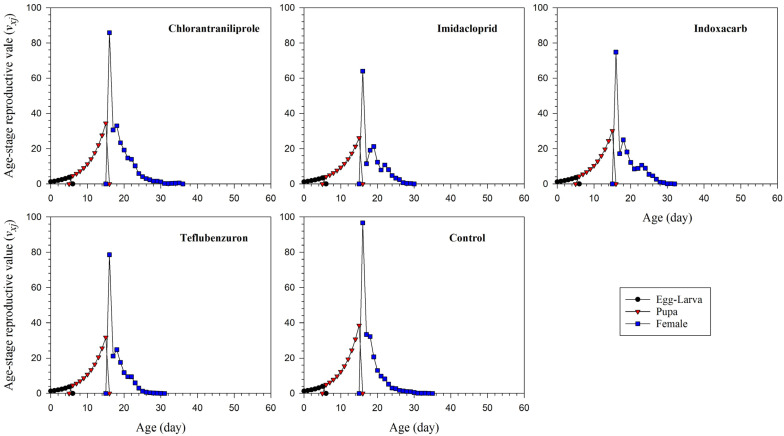
Age–stage reproductive value (*v_xj_*) of *Telenomus busseolae* adults exposed to LC_25_ concentration of four different insecticides and a water control.

The life expectancy (*E*ₓⱼ) and reproductive value (*v*ₓⱼ) of *T. busseolae* varied among treatments (Figure A1 and Figure A2). Life expectancy (*E*ₓⱼ), defined as the expected remaining lifespan (days) of an individual *T. busseolae* at age x and developmental stage j, was highest on the first day of the egg–larval and pupal stages under teflubenzuron and the control. *E*ₓⱼ values under chlorantraniliprole, imidacloprid, indoxacarb, teflubenzuron, and control treatments were 33.3, 24.8, 25.5, 34.9, and 31.9 days for the egg–larval stage, and 27.3, 18.7, 19.5, 28.9, and 25.9 days for the pupal stage, respectively. In the chlorantraniliprole, teflubenzuron, and control treatments, *E*ₓⱼ for adult males and females declined progressively with age, whereas in parasitoids exposed to the LC_25_ of imidacloprid and indoxacarb, *E*ₓⱼ fluctuated but showed an overall decreasing trend (Figure A1).

The reproductive value (*v*ₓⱼ), defined as the contribution of an individual of age *x* and stage *j* to future population growth, also varied among treatments. In all treatments, *v*ₓⱼ was highest on the first day of female parasitoids and declined with age, with minor fluctuations during intermediate stages. The first-day peak was highest in the control (96.6), followed by chlorantraniliprole (85.8), teflubenzuron (78.6), indoxacarb (74.7), and imidacloprid (64.0). In the control and teflubenzuron treatments, *v*ₓⱼ declined gradually over subsequent days, whereas in imidacloprid and indoxacarb it declined sharply immediately after the first day. In chlorantraniliprole, *v*ₓⱼ showed a steep initial drop from the first day, followed by a more gradual, stepwise decline with a minor increase on the second day, and *v*ₓⱼ persisted at low values for several additional days (Figure A2).

### 3.3. Host Exploitation Parameters

Insecticide exposure also affected host exploitation by *T. busseolae* (Table 4). The net parasitism rate (cumulative *P*_0_) was highest in the control (62.1 hosts per individual) and chlorantraniliprole treatment (59.6 hosts per individual), and lowest following imidacloprid exposure (37.9 hosts per individual). However, the transformation rate (*Q*_p_), finite parasitism rate (*ω*), and stable parasitism rate (*ψ*) did not differ significantly among treatments, indicating that the efficiency of host-to-parasitoid conversion remained constant despite variations in overall parasitism levels (Table 4).

### 3.4. Population Growth Projection

**Figure 3:**
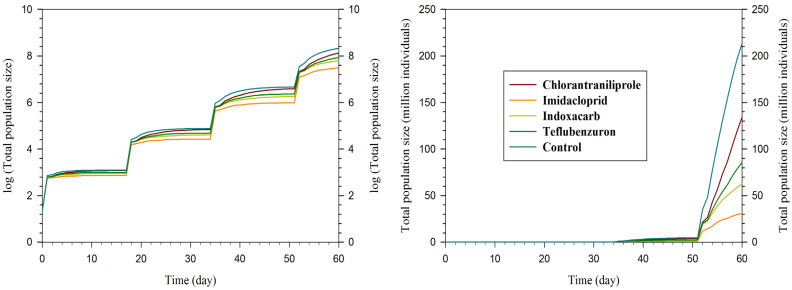
Projection of population growth of *Telenomus busseolae* adults exposed to LC_25_ concentration of four different insecticides and a water control.

Population growth projections initiated with ten pairs of *T. busseolae* under controlled conditions revealed substantial differences among insecticide treatments over 60 days (Figure 3). The control treatment yielded the highest projected population size, reaching approximately 214 million individuals by day 60. Chlorantraniliprole-exposed parasitoids attained the second-highest population size at approximately 134 million individuals, representing 63% of the control population. Teflubenzuron and indoxacarb treatments resulted in intermediate population sizes of approximately 86 million and 63 million individuals, respectively. Imidacloprid produced the lowest population projection, with approximately 31 million individuals at day 60, corresponding to only 14% of the control population size.

The cumulative population sizes diverged markedly after day 45, reflecting the compounding effects of reduced fecundity and longevity parameters observed in insecticide-exposed parasitoids (Figure 3).

## 4. Discussion

In this study, we evaluated the sublethal effects of LC_25_ concentrations of four insecticides registered for maize pests, including the Mediterranean corn stalk borer (*S. nonagrioides*), on the egg parasitoid *T. busseolae* using the age–stage, two-sex life table approach, integrated with host exploitation and population projection analyses. The calculated risk quotient (RQ) values, based on the ratio of field concentrations to LC_50_ toxicity values, were below 50 for all four insecticides, indicating minimal acute toxicity [30]. Nevertheless, life table analysis revealed significant sublethal effects, including reductions in longevity, fecundity, and intrinsic rate of increase. These findings highlight the limitations of relying solely on acute toxicity (LC_50_) and RQ values in risk assessment, as they may fail to capture broader sublethal effects of insecticide exposure (LC_25_) on the egg parasitoid, *T. busseolae*.

Sublethal (LC_25_) exposure to the tested insecticides produced clear, compound-specific effects on the reproductive performance and population dynamics of *T. busseolae*. Imidacloprid exerted the strongest negative impact, significantly reducing total and female fecundity as well as key demographic parameters, including the intrinsic rate of increase (r) and finite rate of increase (*λ*). In contrast, chlorantraniliprole maintained reproductive output comparable to the control, although it showed a tendency to increase the proportion of male offspring. Despite these shifts in sex ratio, female reproductive rate (*R*_0_) remained relatively stable across treatments, whereas male reproductive rate declined under imidacloprid and indoxacarb exposure. Overall, population growth (*R*_0_) closely followed fecundity patterns, with the lowest values recorded under imidacloprid and the highest under chlorantraniliprole and the control. These results indicate that reductions in population growth were driven primarily by decreased maternal reproductive performance rather than by changes in sex allocation. Although *T. busseolae* is an arrhenotokous parasitoid capable of adjusting offspring sex ratio through haplodiploidy [9], this mechanism was insufficient to compensate for the negative effects of sublethal exposure on fecundity and survival. Oviposition duration further supported this pattern, remaining stable under chlorantraniliprole but progressively decreasing under teflubenzuron, indoxacarb, and especially imidacloprid. This reduction likely reflects physiological stress and neurophysiological disruption that limit host exploitation and shorten the effective reproductive period.

Adult longevity and oviposition duration showed clear compound-dependent responses to LC_25_ exposure in *T. busseolae*. Females exposed to chlorantraniliprole and teflubenzuron maintained or slightly extended lifespan relative to the control, whereas indoxacarb and imidacloprid reduced survival and progressively shortened oviposition duration, likely reflecting physiological stress and neurophysiological disruption. Life expectancy (*E*ₓⱼ) and reproductive value (*v*ₓⱼ) were similarly affected, with imidacloprid and indoxacarb showing faster declines compared with chlorantraniliprole, teflubenzuron, and the control. The peak in reproductive value at the onset of oviposition is consistent with the proovigenic strategy of *T. busseolae* [9,43], and its reduction under imidacloprid and indoxacarb further confirms the compound-specific impairment of reproductive capacity. Sublethal exposure to imidacloprid has previously been shown to influence sex allocation in *Nasonia vitripennis* [44], whereas no such effect was observed in the present study, further highlighting species-specific responses to neonicotinoid exposure.

Adult *T. busseolae* exposed to LC_25_ concentrations of the tested insecticides showed no significant reduction in emergence of offspring parasitoids from parasitized hosts, indicating that sublethal exposure did not compromise offspring viability or development. These results suggest that under the tested conditions, sublethal concentrations are unlikely to directly affect offspring emergence. Nevertheless, previous studies have shown that emergence responses in scelionid parasitoids are highly variable and depend on species, insecticide class, concentration, and exposure method. Reduced emergence has been reported in *Telenomus podisi* following exposure to a neonicotinoid–pyrethroid mixture [45], whereas *Telenomus remus* showed no such effect under similar conditions [26]. Similarly, imidacloprid exposure reduced emergence and parasitism in *T. podisi* [46]. In *T. remus*, emergence responses varied with exposure pathway, with surface application generally being less toxic than direct topical exposure [25].

Life table studies assessing sublethal insecticide effects in scelionid parasitoids remain limited, with most research focusing on acute toxicity and a few selected biological traits rather than comprehensive demographic parameters [21,23,25]. Earlier approaches have largely relied on female-based fertility tables, which provide only a partial representation of population dynamics by excluding male contributions and stage differentiation [11,19,26]. The age–stage, two-sex life table used in the present study therefore provides a more comprehensive framework, integrating both sexes and all developmental stages for a more realistic assessment of population performance. The results of the present study indicate that life table responses in scelionid parasitoids depend strongly on species, insecticide class, and exposure level. For example, *T. remus* showed no significant changes in population parameters following exposure to several insecticides at LC_50_ (median lethal concentration) levels [26], whereas in *T. busseolae*, reductions in life table parameters were compound-specific (deltamethrin vs. cyfluthrin) [11]. Similarly, *Trissolcus grandis* exhibited limited sensitivity to field-rate insecticide exposure, with no significant effects on key demographic traits [19]. Collectively, these comparisons indicate that sublethal responses are context-dependent and influenced by multiple interacting factors, including species biology, insecticide properties, concentration, developmental stage, and exposure route.

The offspring sex ratio (female proportion) of *T. busseolae* was significantly affected by parental exposure to LC_25_ concentrations of the tested insecticides. Chlorantraniliprole produced male-biased progeny, whereas indoxacarb and imidacloprid resulted in female-biased offspring, and teflubenzuron produced intermediate sex ratios. These patterns are consistent with the arrhenotokous reproductive biology of the species, which typically generates female-biased progeny under normal conditions [9,43,47]. Despite reduced female proportions, chlorantraniliprole and teflubenzuron maintained higher population growth due to increased fecundity and adult longevity, indicating that maternal performance may play a more important role in shaping demographic outcomes than sex allocation alone. Sex ratio responses to insecticides vary among scelionid parasitoids depending on species, insecticide class, and exposure conditions. For example, no significant effects on sex ratio have been reported for *T. remus* under several insecticide treatments [25,26], and similar results were observed in *T. podisi* exposed to neonicotinoids and pyrethroids [46]. In other egg parasitoids, however, chlorantraniliprole altered the sex ratio of *Trichogramma chilonis* in a resistance-dependent manner, whereas indoxacarb showed no effect [48,49]. In the present study, chlorantraniliprole produced a sex ratio comparable to the control, indicating that species- and compound-specific responses, together with maternal performance, shape population outcomes. These differences are likely related to modes of action: imidacloprid disrupts neural signaling via nicotinic acetylcholine receptors, indoxacarb acts as a sodium channel blocker with delayed activation, and teflubenzuron interferes with chitin synthesis and development, whereas chlorantraniliprole, targeting ryanodine receptors, appeared less disruptive at LC_25_, allowing maintenance of reproductive activity and survival. These mechanistic differences explain the compound-specific life-history responses observed.

Our results suggest that sublethal LC_25_ exposure influenced the host exploitation capacity of *T. busseolae*. Net parasitism was highest in the control and under chlorantraniliprole, intermediate under indoxacarb and teflubenzuron, and lowest under imidacloprid, indicating that imidacloprid may impair host searching and oviposition performance, likely through neurotoxic effects. In contrast, transformation rate, finite parasitism rate, and stable parasitism rates showed no clear differences among treatments, suggesting that LC_25_ exposure did not substantially affect developmental success of parasitized hosts. Thus, differences in parasitism primarily reflect changes in host exploitation efficiency rather than developmental constraints. Although consumption-based analyses using the CONSUME-MSChart approach remain rarely applied to parasitoids [50,51,52], previous studies report comparable patterns. Majidpour et al. [50] observed concentration-dependent reductions in parasitism-related parameters in *Aphidius flaviventris* exposed to a thiacloprid + deltamethrin mixture, while Guo et al. [51] reported that neonicotinoids and other insecticides significantly affected host feeding and killing rates in *Eretmocerus hayati*, despite limited effects on developmental parameters. These findings are consistent with our results for imidacloprid, highlighting that sublethal exposure can strongly impair parasitoid host exploitation even when developmental success remains largely unaffected [50,51,52].

Population projection analyses indicated that LC_25_ exposure may alter population trajectories of *T. busseolae* under laboratory-based simulations. The control population increased most rapidly over the 60-day period, followed by chlorantraniliprole, whereas teflubenzuron and indoxacarb showed intermediate growth and imidacloprid produced the lowest population increase. These projections are derived from life table parameters under controlled laboratory conditions and are intended for comparative purposes rather than direct field prediction. Overall, the results suggest that imidacloprid may reduce population growth and, consequently, biological control potential of *T. busseolae*. Few studies have applied population projection approaches to pesticide-exposed parasitoids. For example, Guo et al. [51] similarly reported reduced population growth and host suppression following sublethal exposure in *E. hayati*, highlighting how cumulative effects across generations may weaken biological control performance of *B. tabaci*.

Because our findings are based on controlled laboratory conditions, future investigations should extend to semi-field and field trials to validate the compatibility of these insecticides with *T. busseolae* in maize pest management systems. Under ecologically relevant conditions, factors such as pesticide residue persistence, plant architecture, and host availability may influence parasitoid responses. Further research should also compare the effects of technical versus formulated imidacloprid, indoxacarb, teflubenzuron, and chlorantraniliprole on *T. busseolae*, quantify insecticide uptake or transfer into parasitoid tissues, and examine host patch exploitation and patch-leaving behavior under sublethal exposure. In addition, investigating gene expression patterns related to host location and parasitism could provide mechanistic insights into observed behavioral changes. These approaches remain largely unexplored and are likely to provide critical insights for designing integrated pest management strategies that maintain compatibility between these insecticides and *T. busseolae*, ultimately enhancing effective management of *S. nonagrioides* in maize agroecosystems.

## Figures and Tables

**Table 1:** Active ingredients, IRAC classifications, trade names and companies, formulations, field rates, field recommended rates, and tested concentrations used in bioassays to estimate LC25 for *Telenomus busseolae*.

Active Ingredient	IRAC Classification	Trade Name and Company	Formulation	Active Ingredient (g/L)	Field Rate (mL/L)	Active Ingredient at Field Application Rate (mg a.i./L)	Field-Recommended Concentration (mL/ha)	Bioassay Test Concentrations (mL/L; Serial Dilutions of Field-Rate Solution)
			Insecticides					
Imidacloprid	Neonicotinoid (4A)	Confidor-BAYER	SC	350	1.75	612.5	350	1.75, 0.875, 0.4375, 0.21875, 0.10937
Teflubenzuron	Benzoylurea (15)	Nomolt Super-BASF	SC	150	2	300	400	8, 4, 2, 1, 0.5
Indoxacarb	Oxadiazine (22A)	Invut-ONC CROPSCIENCE	SC	150	1.5	225	300	1.5, 0.75, 0.375, 0.1875, 0.09375
Chlorantraniliprole	Anthranilic Diamide (28)	Coragen-DUPONT	SC	200	0.75	150	150	1.5, 0.75, 0.375, 0.1875, 0.09375

IRAC = Insecticide Resistance Action Committee. Field application volume was set at 200 L per hectare for the calculation of concentrations.

**Table 2:** Concentration–response statistics for four insecticides with different modes of action for *Telenomus busseolae* based on probit regression, and risk quotients and categories.

Active İngredient	Exposure Duration (h)	Sample Size (n)	Slope ± SE	LC_25_ (mL/L) (95% FL)	LC_50_ (mL/L) (95% FL)	χ^2^, df, *p*	The Risk Quotients (RQ) (FRC/LC_50_ mL/L Basis for the Parasitoid)	RQ (g a.i./ha ÷ LC_50_ mg a.i./L for the Parasitoid)	Risk Category
Chlorantraniliprole	24	721	1.965 ± 0.159	0.398 (0.345–0.449)	0.696 (0.622–0.784)	11.443, 18, 0.875	1.08	0.215	Harmless
Imidacloprid	12	962	0.499 ± 0.049	0.114 (0.078–0.150)	0.440 (0.366–0.529)	13.456, 18, 0.764	3.98	0.796	Harmless
Indoxacarb	12	961	1.887 ± 0.136	0.421 (0.372–0.469)	0.754 (0.681–0.841)	9.514, 18, 0.947	1.99	0.398	Harmless
Teflubenzuron	24	722	1.756 ± 0.143	1.868 (1.601–2.129)	3.493 (3.097–3.976)	12.302, 18, 0.831	0.57	0.115	Harmless

FL: 95% fiducial limits (confidence interval) of the lethal concentrations.

**Table 3:** Life-history traits of *Telenomus busseolae* after exposure to LC_25_ concentrations four insecticides with different modes of action and an untreated control (distilled water).

Treatments					
	Control (Distilled Water (n = 35)	Chlorantraniliprole LC_25_ (n = 28)	Imidacloprid LC_25_ (n = 25)	Indoxacarb LC_25_ (n = 24)	Teflubenzuron LC_25_ (n = 26)
Fecundity (total)	123.1 ± 3.63 a	118.3 ± 5.88 a	72.9 ± 5.65 c	89.9 ± 6.30 b	97.5 ± 7.32 b
Fecundity (Female offspring)	69.8 ± 2.46 a	53.2 ± 2.93 bc	49.9 ± 3.14 c	58.9 ± 1.78 b	56.7 ± 2.93 bc
Fecundity (Male offspring)	53.2 ± 3.59 ab	65.1 ± 5.11 a	23.0 ± 4.55 c	31.1 ± 5.61 bc	40.8 ± 6.01 b
Net reproductive rate *R*_0_ (Female offspring)	34.9 ± 4.36 a	26.61 ± 3.83 a	24.94 ± 3.83 a	29.43 ± 4.33 a	28.34 ± 4.19 a
Net reproductive rate *R*_0_ (Male offspring)	26.6 ± 3.64 a	32.5 ± 5.01 a	11.5 ± 2.78 b	15.5 ± 3.57 b	20.4 ± 4.12 ab
Net reproductive rate *R*_0_ (total)	61.5 ± 7.45 a	59.2 ± 8.42 a	36.4 ± 5.86 b	44.9 ± 7.22 ab	48.7 ± 7.66 ab
Intrinsic rate of increase (*r*)	0.2281 ± 0.0070 a	0.2211 ± 0.0079 ab	0.2032 ± 0.0090 b	0.2130 ± 0.0088 ab	0.2150 ± 0.008 ab
Finite rate of increase λ	1.2557 ± 0.0087 a	1.2475 ± 0.0099 ab	1.2253 ± 0.0119 b	1.2360 ± 0.0196 ab	1.2400 ± 0.0109 ab
Oviposition days	8.6 ± 0.55 a	9.2 ± 0.73 a	3.7 ± 0.74 c	5.7 ± 0.90 bc	6.6 ± 0.75 b
Female longevity (day)	15.9 ± 1.15 b,A	20.6 ± 1.22 a,A	8.8 ± 2.08 c,A	9.4 ± 1.48 c,A	20.3 ± 1.79 a,A
Male longevity (day)	16.0 ± 1.13 a,A	13.9 ± 1.39 a,B	8.7 ± 1.20 b,A	9.5 ± 1.13 b,A	17.4 ± 1.19 a,A
Emergence (%) *	98.6 ± 0.61 a	98.8 ± 0.68 a	97.0 ± 0.73 a	98.9 ± 0.7 a	99.2 ± 0.71 a
Sex ratio (female) (%) *	57.4 ± 2.80 ab	46.7 ± 3.13 a	71.0 ± 3.39 c	72.6 ± 3.31 c	63.9 ± 3.25 bc

Different lowercase letters in each row indicate significant differences between treatments (paired bootstrap test, *p* < 0.05). Different uppercase letters in columns indicate significant differences between male and female longevity (paired bootstrap test, *p* < 0.05). Standard errors (SE) were estimated with 100,000 bootstrap resampling. * Indicates statistical comparison using one-way ANOVA with Tukey HSD test for emergence and sex ratio (female) comparisons within the same row across treatments (*p* < 0.05). n = the number of replicates for each treatment.

**Table 4:** Host egg exploitation (parasitism) of *Telenomus busseolae* after exposure to LC_25_ concentrations four insecticides with different modes of action and untreated control (distilled water).

Parameters	Treatments				
	Control (Distilled Water)	Chlorantriniliprole LC_25_	Imidacloprid LC_25_	Indoxacarb LC_25_	Teflubenzuron LC_25_
Net Parasitism Rate, *P*_0_ (host/individual)	62.1 ± 7.60 a	59.6 ± 8.50 a	37.9 ± 6.14 b	45.5 ± 7.33 ab	49.2 ± 7.76 ab
Transformation Rate, *Q*_p_ (*P*_0_/*R*_0_)	1.0090 ± 0.0017 a	1.0060 ± 0.0035 a	1.0410 ± 0.0231 a	1.0120 ± 0.0026 a	1.0100 ± 0.0029 a
Finite Parasitism Rate, *ω* (host)	1.5870 ± 0.0220 a	1.5665 ± 0.0250 a	1.5384 ± 0.0298 a	1.5451 ± 0.0277 a	1.5531 ± 0.0281 a
Stable Parasitism Rate, *ψ* (host)	1.2637 ± 0.0087 a	1.2562 ± 0.0101 a	1.2553 ± 0.0147 a	1.2494 ± 0.0115 a	1.2505 ± 0.0118 a

Different letters in each row indicate significant differences between treatments (paired bootstrap test, *p* < 0.05). Standard errors (SE) were estimated with 100,000 bootstrap resampling.

## Data Availability

The original contributions presented in this study are included in the article. Further inquiries can be directed to the corresponding author.
